# Notch Signaling Regulates the Chondrogenic Potential of Both Articular Chondrocytes and Their Progenitors During Expansion

**DOI:** 10.1093/stmcls/sxad031

**Published:** 2023-04-21

**Authors:** Anastasiia D Kurenkova, Lei Li, Anna P Usanova, Xiaogang Feng, Baoyi Zhou, Andrei A Nedorubov, Alexey V Lychagin, Andrei S Chagin

**Affiliations:** Institute for Regenerative Medicine, I.M. Sechenov First Moscow State Medical University, Moscow, Russia; Department of Internal Medicine and Nutrition, Institute for Medicine, Gothenburg University, Gothenburg, Sweden; Department of Physiology and Pharmacology, Karolinska Institutet, Stockholm, Sweden; Institute for Regenerative Medicine, I.M. Sechenov First Moscow State Medical University, Moscow, Russia; Department of Microbiology, Tumour and Cell Biology, Karolinska Institutet, Stockholm, Sweden; Department of Physiology and Pharmacology, Karolinska Institutet, Stockholm, Sweden; Center for Preclinical Studies, Institute of Translational Medicine and Biotechnology, I.M. Sechenov First Moscow State Medical University, Moscow, Russia; Department of Traumatology, Orthopedics, and Disaster Surgery, I.M. Sechenov First Moscow State Medical University, Moscow, Russia; Department of Internal Medicine and Nutrition, Institute for Medicine, Gothenburg University, Gothenburg, Sweden; Department of Physiology and Pharmacology, Karolinska Institutet, Stockholm, Sweden

## Abstract

Articular cartilage has a limited capacity for self-repair and clinical approaches to cartilage regeneration are needed. The only such approach developed to date involves an expansion of primary autologous chondrocytes in culture, followed by their reimplantation into a cartilage defect. However, because of the formation of fibrocartilage instead of hyaline cartilage, the outcome is often not satisfactory. It happens due to the de-differentiation of chondrocytes during the expansion step. Indeed, articular chondrocytes are non-proliferative and require partial or complete dedifferentiation before actively proliferating. In recent years stem/progenitor cells in articular cartilage (artSPCs) have been described. These cells maintain their own population and renew articular cartilage in sexually mature mice. artSPCs can, theoretically, be superior to chondrocytes, for repairing damaged cartilage.

Accordingly, here, we searched for conditions that allow rapid expansion of both artSPCs and chondrocytes with simultaneous preservation of their ability to form hyaline cartilage. Among the modulators of Wnt, Notch, and FGF signaling and of cell adhesion screened, only fibronectin and modulators of the Notch pathway promoted the rapid expansion of artSPCs. Surprisingly, both inhibition and activation of the pathway had this effect. However, only inhibition of Notch during expansion facilitated the chondrogenic potential of both artSPCs and primary chondrocytes, whereas activation of this pathway abrogated this potential entirely. This effect was the same for murine and human cells. Our present observations indicate that Notch signaling is the major regulator of the chondrogenic capacity of both artSPCs and chondrocytes during their expansion.

## Graphical abstract

**Graphical Abstract:**
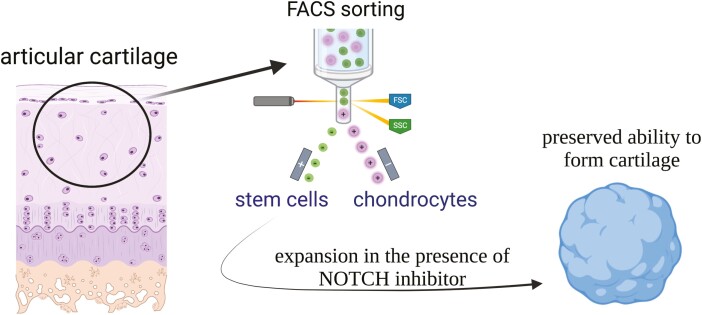

**Significance Statement**

The only stem-cell-based approach to repair articular cartilage to date involves the expansion of primary autologous chondrocytes in culture, followed by their re-implantation into a cartilage defect. However, dedifferentiation of chondrocytes and chondroprogenitors during expansion causes the formation of fibrocartilage. We found that inhibition of Notch pathway during this expansion step preserves the chondrogenic potential in both chondroprogenitors and primary chondrocytes obtained either from mice or humans. This simple and compatible with current clinical routine modification of the expansion protocol will hopefully improve the current clinical approaches to cartilage regeneration.

## Introduction

Articular cartilage, an extensively hydrated, avascular, and aneural tissue that facilitates skeletal articulation, consists morphologically of a superficial, middle (or intermediate), and deep zone. The superficial zone contains small flatten cells expressing high levels of lubricin [encoded by proteoglycan 4 (Prg4)] and tenascin-C and low levels of aggrecan.^1,2^

Currently, the only cell-based approach approved for clinical repair of focal cartilage defects, first proposed by Brittberg et al. (1994),^3^ involves an expansion of autologous chondrocytes in culture followed by their re-implantation without (ACI) or with (MACI) matrix. Since this treatment results in a joint that functions properly for many years, thereby delaying the necessity for joint-replacement surgery, it is of particular value for young adults.^4^ A problem often encountered with ACI/MACI is the formation of fibrocartilage rather than the more durable hyaline cartilage,^5^ due to the progressive dedifferentiation of chondrocytes during their expansion in monolayer culture.^6–8^ This dedifferentiation can be reversed to a certain extent,^8–10^ but the outcome remains far from ideal.

On the basis of their capacity to form colonies in vitro, expand extensively and contribute to the development of various tissues in vivo, it has been proposed that progenitor cells in articular cartilage are mainly located in the superficial zone.^11^ Definitive evidence for this was provided by the demonstration by genetic tracing that in Prg4-driven CreERt mice coupled with the R26R-LacZ reporter strain, Prg4-positive cells, located on the surface of the cartilage, can generate chondrocytes in articular cartilage.^12^ In fact, the entire articular cartilage of adult mice is generated by the progeny of Prg4-positive cells,^13^ which also contribute to the healing of focal cartilage defects.^14^

It is noteworthy that these progenitor cells undergo renewal via symmetric division and express the stem cell markers CD73 (ecto-5ʹ-nucleotidase),^13^ CD105 and CD34.^15^ Collagen XXII is probably a constituent of the niche for these cells located at the surface of articular cartilage.^16^ Accordingly, these features allow us to define the superficial chondroprogenitors as articular stem and progenitor cells (artSPCs)^17^ and we hypothesize that artSPCs may provide a better cellular source for ACI/MACI than chondrocytes.

In general, signaling pathways that play pivotal roles in development participate in the regulation of stem cells.^18,19^ The pathways associated with joint formation include Wnt/b-catenin, hedgehog, FGF, BMP, and TGFb (reviewed in reference^20^) and have been implicated in the maintenance of stem cells in various compartments of bone (reviewed in reference^17^).

Wnt signaling initiates the formation of a joint^21^ and overexpression of β-catenin soon after birth results in the thickening of the superficial zone.^22,23^ FGF-2, -4, -10, -13, and -18 as well as the FGF receptor2 (FGFR2) and FGFR3 are expressed at high levels in the interzone, which later develops into the joint.^24^ The outcome of Notch signaling, a highly conserved pathway involved in determining the fates of cells, including the self-renewal, proliferation, differentiation, and apoptosis of stem/progenitor cells, is dependent on the environment and past history of the cell in question. Relatively high levels of Notch1, Delta1, Jagged1, and Jagged2 are present in the superficial zone of immature bovine articular cartilage,^25^ indicating that these factors are likely to help regulate the formation, maintenance, and homeostasis of artSPCs.

In addition to such morphogens, regulation of the artSPCs is likely to involve mechanical forces and the surrounding matrix. Indeed, movement during early development facilitates the appearance of the synovial cavity and the establishment of the articular joint.^26^ Moreover, in direct response to sheer stress, artSPCs increase their production of lubricin through a process involving prostaglandin E2 and the transcription factor CREB.^27^

Furthermore, in contrast to articular cartilage, the matrix of the superficial layer, which houses the artSPCs, contains collagen types I and XXII. In addition, artSPCs adhere faster to fibronectin^11^ and propagate well on plastic coated with this protein.^28,29^ As a consequence of this seminal work by Dowthwaite et al. (2004), plastic coated with fibronectin is now widely used to isolate and culture cells derived from the superficial layer of articular cartilage.^25,30–32^

In this current investigation, we attempted to optimize conditions for the expansion of artSPCs without compromising their chondrogenic potential. In this context we explored the potential usefulness of the Wnt, FGF, and Notch signaling pathways, utilizing fibronectin as a positive control for comparison.

## Results

### artSPCs Cells Form Clones and Undergo Multi-Lineage Differentiation

**Figure 1.**
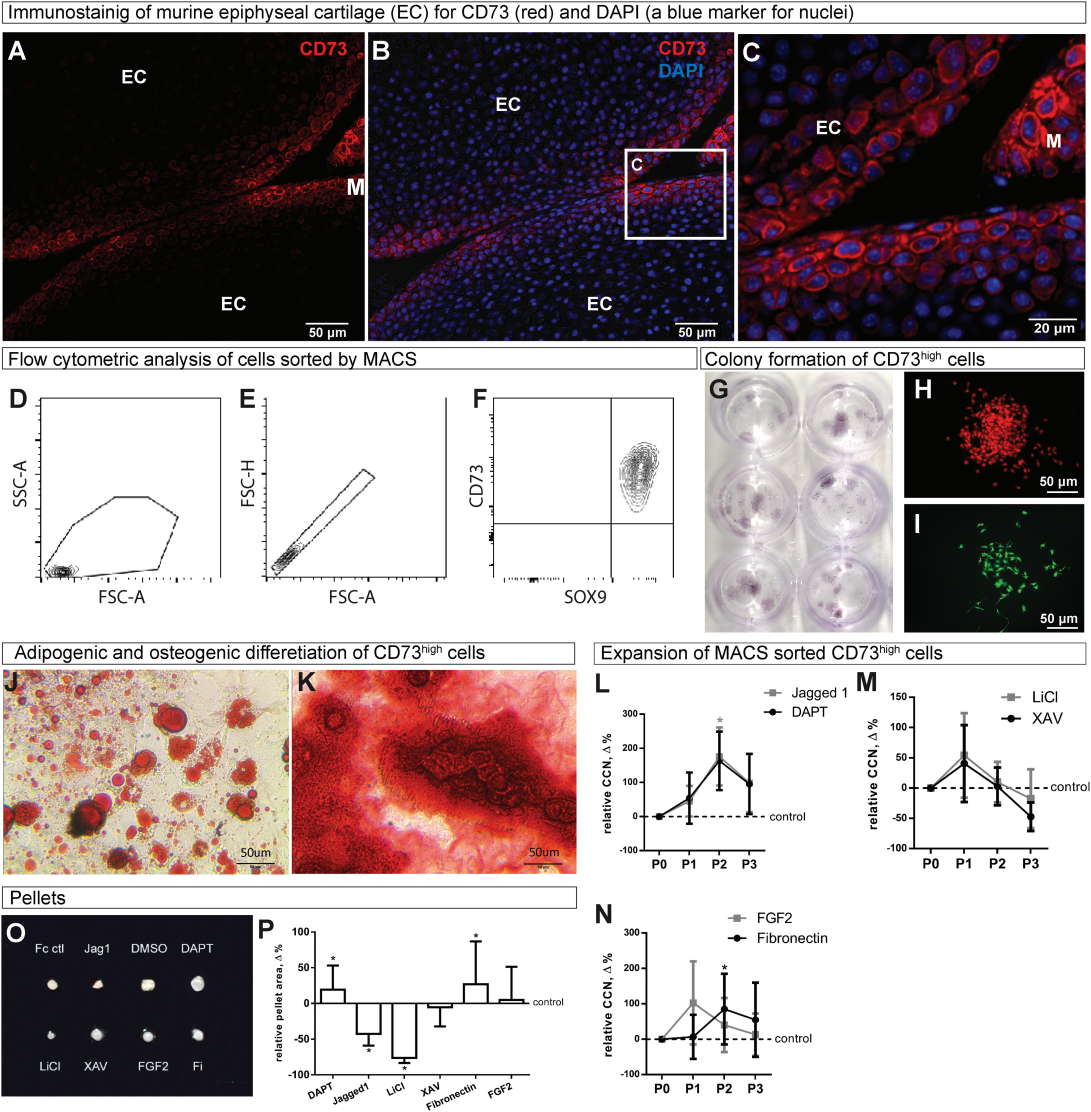
Properties and expanding conditions for murine artSPCs. (**A**, **B**) The pattern of CD73 expression in the articular cartilage of 6-day-old mice. The square in B is magnified in (**C**). (**D-F**) Flow cytometric analysis of CD73^high^ cells separated by MACS confirmed their expression of both Sox9 and CD73. (**G-I**) Formation of colonies by CD73^high^ cells obtained from Prg4CreERT:R26-Confetti mice and visualized by hematoxylin staining (G) or by direct fluorescence of (**H**) RFP or (**I**) GFP. H and I exemplify clonal colonies made by a single original cell harboring one Confetti allele after recombination. (**J**) Adipogenic (stained with Oil red O) and (K) osteogenic (stained with alizarin red) differentiation of CD73^high^ cells isolated by MACS and cultured in the corresponding medium for 21 days. (**L-N**) CD73^high^ cells were MACS-isolated, and their expansion rate was assessed in various conditions. The cumulative number of cells (CNN) after 3 passages in cultures containing (L) DAPT or Jagged1; (M) XAV or LiCl; or (N) FGF2 or fibronectin were compared to those of corresponding control cells. (**O**) The formation of chondrogenic pellets of different sizes from the same number of cells after 21 days of culture in the presence of different factors. (**P**) The areas of the pellets shown in (O) were determined by ImageJ and normalized to the corresponding controls. *P < .05 is a comparison to the corresponding control group (DMSO in the case of DAPT, XAV, and LiCl; Fc for Jagged1; and PBS for Fibronectin and FGF2). ANOVA with the post hoc Dunnett’s test was used to access differences between more than 2 groups (ie, DMSO, DAPT, XAV, and LiCl; PBS, Fibronectin, and FGF2) and Student’s unpaired *t*-test to compare Jagged1 with the Fc control. In all cases cells were pooled together from 5 to 6 mice, 3 independent experiments were performed, and the values shown are the means and SDs of the 3 values obtained. Representative images and gating strategy are shown in A-K and O. EC, epiphyseal cartilage; M, meniscus in A-C; FSC-A= forward scatter area; FSC-H, forward scatter height; SSC-A, side scatter area.

Previously, we demonstrated that murine artSPCs, which generate chondrocytes in vivo, express the mesenchymal stem cell marker CD73.^13^ In agreement with this earlier finding, most CD73^high^ cells detected here in the superficial zone of articular cartilage, with a few located at the surface of the meniscus (Fig. 1A-1C). Thereafter, articular cartilage was isolated surgically and digested briefly with collagenase, followed by isolation of CD73^high^ cells by magnetic-activated cell sorting (MACS) and subsequent analysis of their purity on the basis of expression of both CD73 and Sox9, as determined by FACS (Fig. 1D-1F). We found that 95.5% of cells isolated were CD73^high^/Sox9+, 0.66% CD73^high^/Sox9−, and only 3.79% have low CD73 level, including 3.41% CD73^low^/Sox9+ and 0.38% CD73^low^/Sox9−. Thus, the specificity of the MACS procedure and the purity of the cells obtained were confirmed.

To verify the stem cell properties of the cells obtained, colony formation from diluted solutions of cells was assayed. After 12 days of culture distinct colonies containing >60 cells each were observed (Fig. 1G). To determine whether these colonies originated from individual cells, CD73^high^ cells were isolated from Prg4-CreERt2:R26R-Confetti mice pulsed with tamoxifen 3-4 days earlier. Mono-clonal colonies were detected (Fig. 1H, 1I), thereby confirming the ability of individual cells to form colonies.

Finally, to explore the capacity of the superficial cells obtained to differentiate into different mesenchymal lineages, CD73^high^ cells were placed immediately after MACS into either adipogenic or osteogenic culture medium for 21 days, following which numerous adipocytes or osteoblasts, respectively, could be identified (Fig. 1J, 1K).

Altogether, these experiments are in good agreement with previous observations^11–13^ and confirm that the CD73^high^ cells obtained are artSPCs.

### The Notch Pathway is Among the Most Potent Stimulators of artSPCs Proliferation

We next explored the influence of Notch, FGF, and Wnt signaling on the expansion of artSPCs during 3 passages (as shown in Supplementary Fig. S1A), utilizing fibronectin-covered plastic as a positive control. Cell growth was promoted most extensively by fibronectin (a 76-fold increase from the starting number, 54.8% greater than control cells), as well as by both an activator (Jagged-1, 121-fold increase from the starting number, 98.6% greater than control) and inhibitor (DAPT, 71-fold increase, 95.4% greater than control) of Notch signaling (Fig. 1L, 1M). FGF2 promoted cell expansion transiently (Fig. 1N); while activation of the Wnt/b-catenin pathway by LiCl had no effect and inhibition of this same pathway by XAV attenuated proliferation (Fig. 1M). Thus, fibronectin, DAPT, and Jagged1 resulted in the most extensive expansion, with both activation and inhibition of the Notch pathway exerting similar effects.

When we subsequently characterized the expression of various markers of artSPCs (Prg4, DKK3, GDF5, CD73), chondrocytes (Sox9, collagen II), and osteoblasts (collagen I) by the cells after 3 passages, the pattern with respect to artSPCs was inconsistent. FGF2 resulted in high expression of Prg4 and low expression of DKK3 (Supplementary Fig. S1B, S1C); LiCl and fibronectin both elevated the expression of Gdf5 (Supplementary Fig. S1D); whereas DAPT and Jagged1 reduced CD73 expression (Supplementary Fig. S1E).

At the same time, Jagged-1 upregulated the osteoblast marker collagen I (Supplementary Fig. S1F) and downregulated both markers of chondrocytes, collagen II and Sox9 (Supplementary Fig. S1G, S1H). In contrast, inhibition of Notch signaling with DAPT decreased the expression of collagen I (Supplementary Fig. S1F) and elevated the levels of both collagen II and Sox9, albeit the latter not to a significant extent (Supplementary Fig. S1G, S1H). Cells expanded on fibronectin influenced the levels of collagens I and II and Sox9 to an extent similar to DAPT (Supplementary Fig. S1F-S1H). Analysis of the expression of Notch transmembrane receptors (Notch1, Notch2, Notch3, and Notch4), as well as of the Hes1 and Hey1 target genes confirmed the presence of the necessary receptors and the expected cellular response to Jagged 1 (Supplementary Fig. S1I-S1N).

These observations indicate that among the agents examined, DAPT and fibronectin preserve chondrogenic potential most effectively during cell expansion.

### During Expansion, the Chondrogenic Potential Is Controlled by the Notch Pathway

Thereafter, we set to explore the ability of cells expanded as above (see Supplementary Fig. S1A) to form hyaline cartilage. Expansion conditions were found to exert a clear impact on the ability of the expanded artSPCs to form hyaline cartilage. Cells, expanded in the presence of DAPT or on fibronectin-coated dishes formed the largest cartilage pellets (Fig. 1O, 1P) whereas expansion in the presence of Jagged1 or LiCl resulted in significantly smaller pellets (Fig. 1O, 1P).

**Figure 2.**
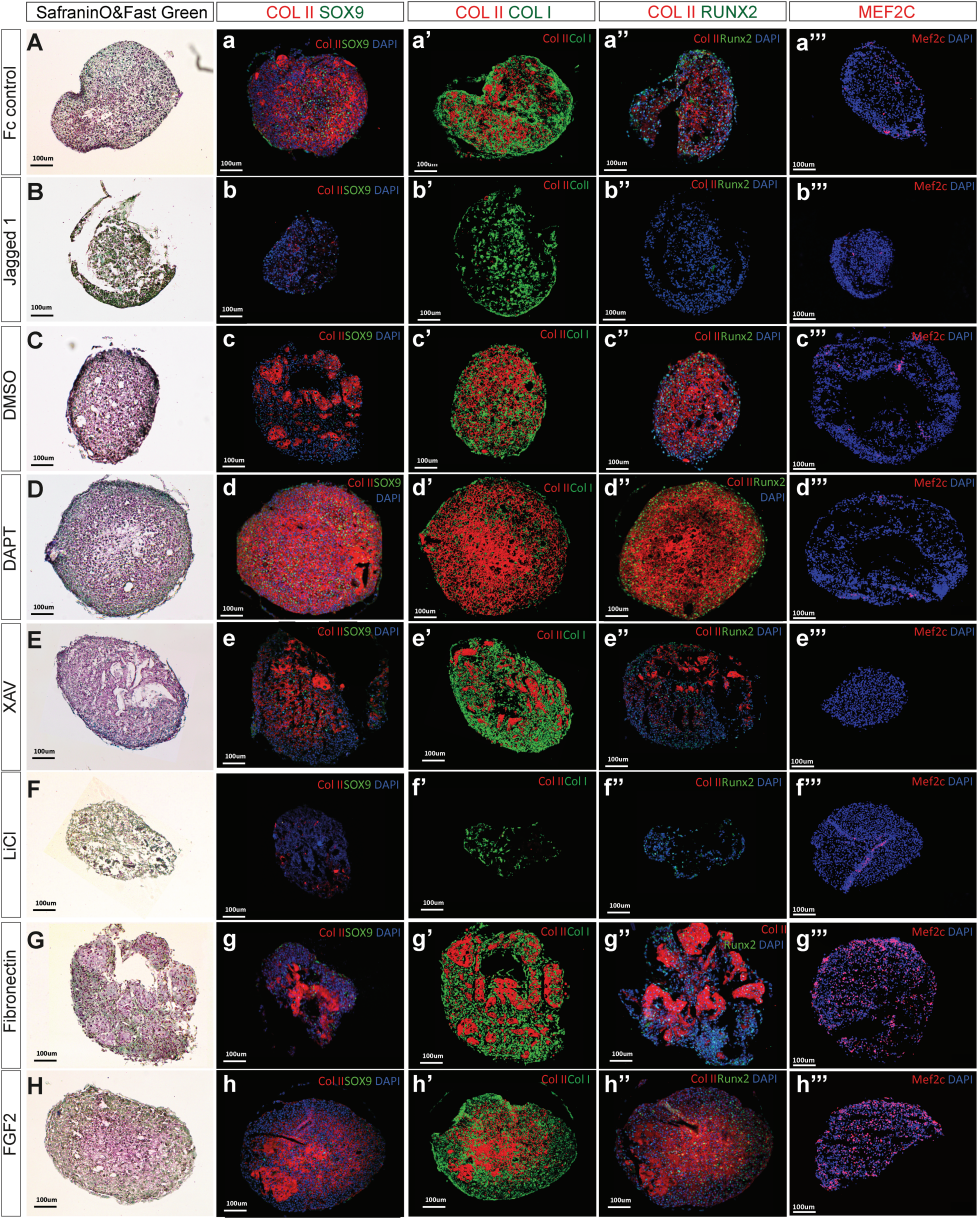
Characterization of cartilage formed by murine CD73^high^ cells expanded in the presence of various factors. (**A-H**) Staining of Safranin O and Fast green was used to visualize cartilage and proteoglycan content. Immunodetection of (****A**ʹ**-H**ʹ**) collagen type II (Col II, red) and the SOX9 transcription factor (green), (**A″-H″**) Col II and collagen type I (green, Col I), (****A**″**ʹ-H**″**ʹ****) Col II and the Runx2 transcription factor (green), and (**A″″-H″″**) the Mef2c transcription factor in combination with nuclei being visualized by DAPI (blue). All images were captured using the same setting and processed with Imaris software. Representative images of 3 independent experiments are shown and cells for each experiment were collected from 5 to 6 mice.

Staining with SafraninO/Fast Green revealed that most proteoglycans were observed in the matrix deposited by artSPCs expanded in the presence of DAPT, DMSO (the vehicle for DAPT), or the Wnt-pathway inhibitor XAV (Fig. 2A-2H). Expansion on fibronectin-coated plastic resulted in small patches that stained positive for proteoglycans in subsequent pellet culture (Fig. 2G). FGF2 resulted in the typical chondrogenic pellets with only proteoglycans deposited in the center (Fig. 2H) while those expanded in the presence of Jagged 1 or LiCl did not deposit any visible proteoglycans in their produced matrix in subsequent pellet culture (Fig. 2B, 2F).

For further characterization of the obtained pellets, we used Sox9 to mark chondrocytes and chondroprogenitors, collagen II to mark mature chondrocytes, collagen I, which marks osteoblasts as well as fibrocartilage, and Runx2, a master transcription factor for osteolineage differentiation but also marks hypertrophic chondrocytes. The highest levels of Sox9 and Collagen II, and the lowest levels of collagen I and Runx2 were observed in the case of cells expanded in the presence of DAPT (Fig. 2Dʹ-2D″″). Moreover, this same group displayed the most homogenous distribution of collagen II (Fig. 2Dʹ-D″ʹ), in contrast to several other groups, including the controls and those expanded in the presence of FGF2 and fibronectin, where this distribution was uneven. In these latter cases, surrounding cells expressed collagen I, suggesting that only a portion of the artSPCs retained their chondrogenic potential during an expansion (Fig. 2Aʹ-2A″″, 2Gʹ-2G″ʹ, 2Hʹ-2H″ʹ). Expansion of artSPCs in the presence of either FGF2 or fibronectin gave rise to a large number of Mef2c-positive cells, indicative of chondrocyte hypertrophy (Fig. 2G″″, 2H″″). Interestingly, artSPCs expanded in the presence of Jagged1 did not differentiate into chondrocytes at all (Fig. 2Bʹ-2B″″) and the associated pellets expressed collagen I abundantly (Fig. 2B″) but no Runx2 or Mef2c (Fig. 2B″ʹ and B″″), suggesting that they contained fibroblast-like cells.

These findings demonstrate clearly that the conditions used for expansion exert an impact on the subsequent chondrogenic potential of artSPCs, with Notch signaling playing the predominant role in this connection.

### Modulation of Notch Activity Influences Chondrocytes and artSPCs in a Similar Manner

Analysis by FACS did not reveal 2 distinct populations of cells containing different levels of CD73, but rather a continuum of cells containing low to high levels (Supplementary Fig. S2A-S2D), which theoretically may reflect a gradual loss of CD73 expression during differentiation of artSPCs toward chondrocytes. When we defined CD73^high^ and CD73^low^ populations by gating as shown in Supplementary Fig. S2C and S2D, the morphology of these sub-populations appeared to differ, as reflected in their scattering properties (Supplementary Fig. S2A).

**Figure 3.**
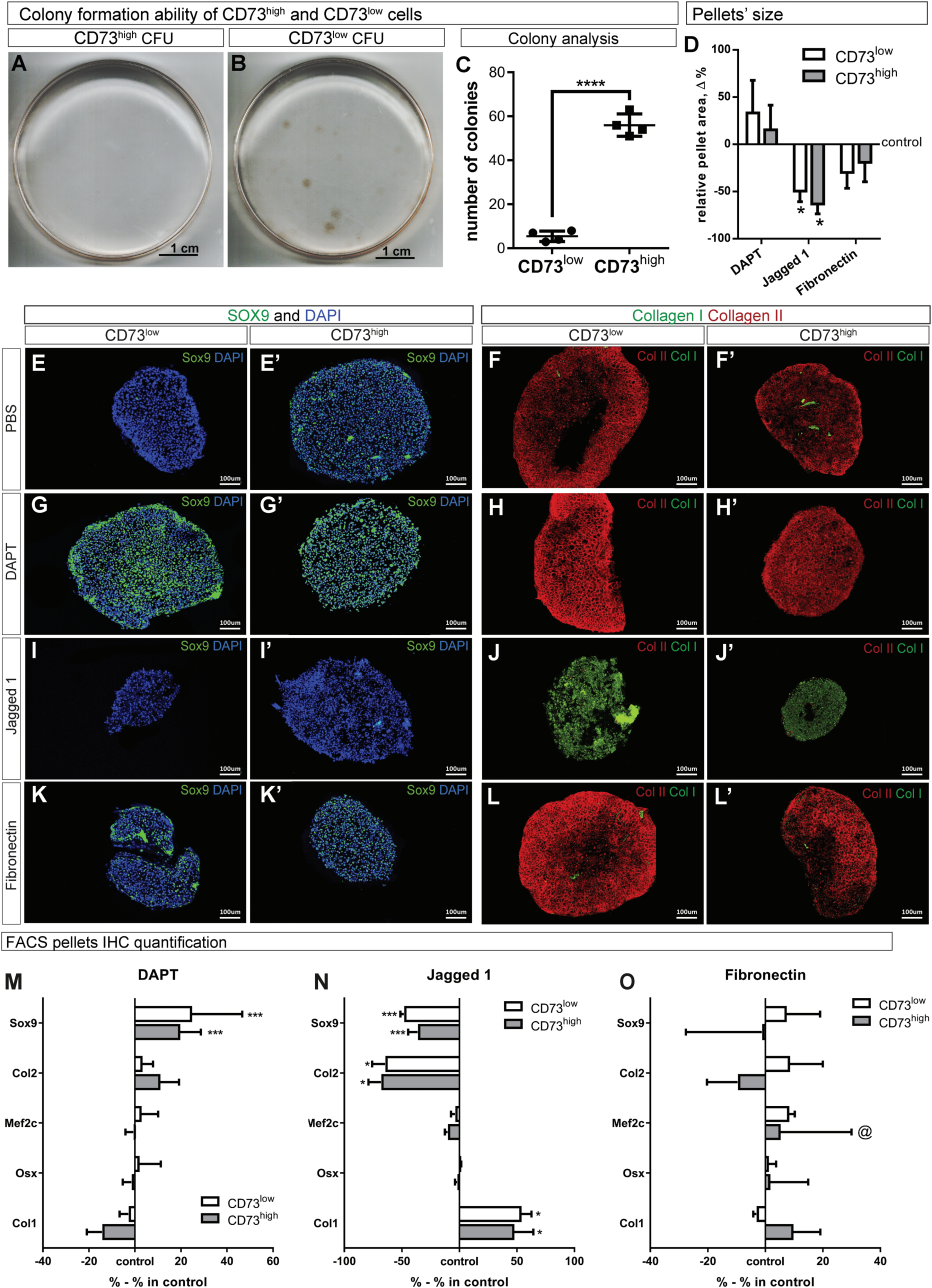
Comparison of colony and cartilage formation by murine artSPCs and chondrocytes following expansion under various conditions. Colony formation by (**A**) CD73^low^ and (**B**) CD73^high^ cells stained with hematoxylin and (**C**) quantification thereof. The chondrogenic capacity after expansion under various conditions was assessed by the formation of cartilage pellets. (**D**) Quantification of pellet size and immunostaining of pellets for (**E-K**ʹ) Sox9 (green) and (**F-L**ʹ) collagens I (green) and II (red) (Col I and Col II). Nuclei were stained blue with DAPI. (**M-O**) Quantification of markers for chondroprogenitors (Sox9), chondrocytes (Col II), hypertrophic chondrocytes (Mef2c), osteoprogenitors (Osterix, Osx), and osteoblasts/fibroblasts (Col I) following expansion in the presence of (M) DAPT, (**N**) Jagged 1 or (O) fibronectin. The values are presented relative to the corresponding control values. Representative images of 5 independent experiments are shown. C: ****P < .0001 as determined by the *t*-test; D, **P-R**: ^@^*P < .*1; *P < .05; ***P < .001 compared to the corresponding control, as determined by ANOVA with post hoc Tukey’s test. *n*=5 in all cases.

As expected, artSPCs (CD73^high^) formed numerous colonies, whereas chondrocytes (CD73^low^) did not (Fig. 3A, 3B). Surprisingly, both populations of cells differentiated toward the adipogenic and osteogenic lineages to a highly similar extent (Supplementary Fig, S3A-S3D). To directly prove that chondrocytes can differentiate into osteoprogenitors and adipocytes, chondrocytes expressing collagen type II were labeled genetically by pulsing Col2CreERT;R26-Confetti mice on postnatal day 2 with tamoxifen and subsequently isolating Confetti-positive cells from surgically dissected cartilage by FACS. Upon appropriate differentiation, both osteoprogenitors (Osx-positive) and adipocytes (perilipin1-positive) cells were detected among the genetically labeled cells (Supplementary Fig. S3E-S3P). This observation aligns well with the extensive plasticity of hypertrophic chondrocytes in vivo observed recently.^33^

Expansion of either artSPCs or chondrocytes in the presence of the Notch inhibitor preserved more chondrogenic potential in comparison with the control cells or fibronectin-expanded cells (Fig. 3E-3Lʹ). In contrast, Notch activation by Jagged1 during expansion totally eliminated the chondrogenic potential of both cell types (Fig. 3I-3Jʹ). Collagen type I, a marker of fibroblasts/osteoblasts, was not detected in cultures expanded in the presence of DAPT (Fig. 3H, Hʹ), but was abundant after culturing in the presence of Jagged1 (Fig. 3J, Jʹ).

Quantification of these data (Fig. 3M-3O), along with markers of chondrocyte hypertrophy (Mef2c) and osteogenic differentiation (Osterix, Osx) (Supplementary Fig. S2N-S2Oʹ), confirmed that expansion in the presence of the Notch inhibitor preserved the chondrogenic potential of both artSPCs and chondrocytes most effectively, without any adverse effect on cell hypertrophy or trans-differentiation toward the osteo-lineage. Interestingly, no Osx was detected after expansion in the presence of Jagged1 (Supplementary Fig. S2M and S2Mʹ), which, coupled with the abundant expression of collagen type I in this case, indicated dedifferentiation of artSPCs and chondrocytes to fibroblast-like cells. The size of the pellets obtained from both artSPCs and chondrocytes was enhanced by DAPT only, although not to a significant extent (Supplementary Fig. S2G). We noted that isolation by FACS damaged artSPCs somewhat, with these cells appearing morphologically unhealthy immediately after sorting and during the first passage (not shown), which may explain their moderate rate of expansion (Supplementary Fig. S2E, S2F) in comparison to MACS-sorted cells (Fig. 1M).

Thus, the Notch pathway appears to be a major regulator of the chondrogenic potential of both chondrocytes and artSPCs. Inhibition of this pathway during expansion preserves the ability of both these types of cells to form hyaline cartilage, whereas activation of this pathway causes a complete loss of their chondrogenic potential.

### Inhibition of Notch Signaling Preserves the Chondrogenic Potential of Human Articular Cartilage Cells

**Figure 4.**
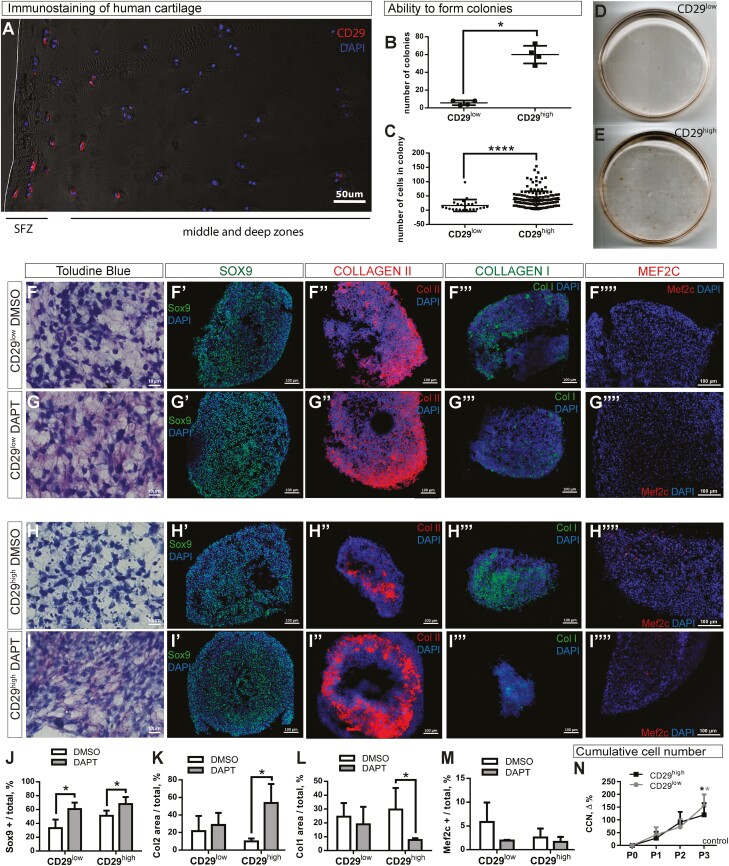
Identification and culturing of human artSPCs and chondrocytes. (**A**) Identification of CD29 as a marker for human superficial cells by immunostaining. (**B-E**) Colony formation by CD29^low^ and CD29^high^ cells. (**F-I**) Toluidine blue staining and immunostaining for (****Fʹ**-I**ʹ****) Sox9, (**F**″-**I**″) collagen type I, (****F**″**ʹ**-I**″**ʹ**) collagen type I, and (**F″″-I″″**) Mef2c in pellets from CD29^low^ and CD29^high^ cells expanded in the presence of DAPT or vehicle (DMSO). Cell nuclei were stained blue with DAPI. Quantification of (**J**) Sox9, (**K**) collagen type II, (**L**) collagen type I, and (**M**) Mef2c in these same pellets. (**N**) Cumulative cell number (CCN) relative to the corresponding controls. Representative images of 5 independent experiments. *P < .05, ****P < .0001, as determined by the unpaired *t*-test. *n* = 5 patients in all cases.

To explore the potential clinical relevance of the observations above, we investigated the effect of inhibiting Notch signaling on the rate of expansion and chondrogenic potential of human articular cartilage cells. CD73 turned out not to be a specific marker for human superficial cells (not shown). Accordingly, we have tested a panel of various surface antigens (CD73, CD29, CD44, CD90, CD105, and CD146) to identify the one, which can selectively label the superficial cells in human articular cartilage. This search revealed that CD29 is a specific marker for human superficial cells, flat in their morphology, and does not label underlying round chondrocytes (Fig. 4A). Sorting by FACS resulted in 2 distinct populations of cells, expressing CD29 at high levels (CD29^high^) and essentially lacking CD29 (CD29^low^) (Supplementary Fig. S4A-S4D), with the former producing significantly more colonies of larger size (Fig. 4B-4E). Both populations underwent osteogenic (Supplementary Fig. S4E-S4F) and adipogenic differentiation (Supplementary Fig. S4G-S4H), in a manner similar to murine chondrocytes and CD73^high^ murine art SPCs.

Inhibition of Notch signaling during cell expansion elevated the chondrogenic potential, ie, increased the number of Sox9-positive cells in the pellets obtained from either CD29^low^ or CD29^high^ cells (Fig. 4Fʹ-4Iʹ, 4J) while the levels of collagen type II were increased only in artSPCs (Fig. 4F″-4I″, 4K). Importantly, expansion in the presence of DAPT reduced the amount of collagen type I in both types of pellets (Fig. 4F″ʹ-4I″ʹ, 4L), suggesting that de-differentiation during the culture period was inhibited. The scarcity of Mef2c in these pellets (Fig. 4F″″-4I″″, 4M) indicated a lack of hypertrophic differentiation. As in the case of murine chondrocytes and artSPCs, the presence of DAPT resulted in an expansion of human chondrocytes and artSPCs that was almost 2.5-fold as great as with control cultures (Fig. 4N).

Thus, inhibition of Notch signaling during the expansion of human articular chondrocytes and especially chondroprogenitors preserves their chondrogenic ability to form cartilage-like pellets afterward and can potentially lead to a better cartilage formation upon reimplantation of expanded cells in such therapies as ACI and MACI.

## Discussion

In the current investigation we show that articular stem cells (artSPCs) from both mice and humans can be successfully expanded in vitro and, in addition, identified conditions that facilitate both the expansion of these cells and simultaneously preserve their chondrogenic potential. Our findings reveal that Notch signaling is a master regulator of chondrogenic potential during the expansion of artSPCs, with activation of this pathway resulting in complete loss of their ability to make hyaline cartilage and inhibition preserving this ability. We also demonstrate that the Notch pathway regulates the chondrogenic potential of cultured primary articular chondrocytes in a similar manner.

At present, treatment with primary autologous articular chondrocytes (eg, involving MACI) is the only therapy approved by the FDA for cartilage regeneration. However, these cells expand slowly and undergo dedifferentiation during this expansion. To overcome these problems, we explored here the expansion and chondrogenic potential of artSPCs for potential alternative use in cartilage regeneration.

These cells generate the entire articular cartilage during postnatal life and express several markers of MSCs, including CD73.^13,34^ Murine artSPCs, isolated on the basis of their high-level expression of CD73, form colonies and, like artSPCs isolated by adhesion to fibronectin, are multi-potent, capable of differentiating into 3 mesenchymal lineages, osteoblasts, adipocytes, and chondrocytes.^11,32,35,36^ Interestingly, CD73, identified in our laboratory as a surface marker for epiphyseal stem cells^13^ in both mice and rats (not shown), proved not to be a specific marker for human artSPCs. Instead, we found that human artSPCs express specifically the surface marker CD29. It is noteworthy that human CD29^high^ cells form colonies and differentiate into osteoblasts, adipocytes, and chondrocytes in a manner similar to murine CD73^high^ cells. The lack of evolutionary conservation of surface markers for other stem and progenitor cells, such as MSCs and HSCs, is well documented^37–39^ and, therefore, not very surprising. CD29 is a β1 subunit of α5β1 integrin, a classical receptor for fibronectin, and all human superficial cells selected by high adherence to fibronectin were reported to be positive for CD29.^30^ CD73 is a common name for 5ʹ-nucleotidase (gene *NT5E*), a common marker for MSCs. CD73 converts mononucleotides to nucleosides, preferentially adenosine monophosphate (AMP) to adenosine, a ligand for adenosine receptors. Both CD73 and adenosine receptors participate in cartilage homeostasis and OA development.^40,41^ Mice and humans differ substantially concerning OA development, which is attributed to differences in posture, weight, and thickness of the articular cartilage.^42^ Inflammation plays a very important role in OA^43^ and mice differ substantially in their inflammatory response from humans.^44^ Altogether, it results in low clinical predictability of mouse models in relation to drug development for OA therapy.^45,46^ Whether the expression of different markers and disease-associated genes by artSPCs contributes to this low predictability remains to be addressed.

Nevertheless, artSPCs can be isolated from both humans and mice and may theoretically allow more efficient MACI. However, their rates of and optimal conditions for expansion, as well as their ability to retain chondrogenic potential during expansion were previously unclear and, accordingly, have been characterized here. Since expansion *ex vivo* is an important step in the MACI procedure,^47^ we first screened for and found 4 conditions that facilitate the expansion of artSPCs, ie, both activation and inhibition of Notch signaling and expansion in the presence of FGF2 or on plastic covered with fibronectin.

However, further analysis revealed that chondrogenic potential is preserved only when these cells are expanded in the presence of the Notch inhibitor, DAPT, whereas activation of this pathway completely abrogates the ability to make hyaline cartilage. Moreover, neither FGF2 nor fibronectin exerts a significant impact on chondrogenic potential, and a certain degree of de- and/or trans-differentiation, similar to that observed in control cultures, is observed under these conditions. Partial loss of chondrogenic potential is probably the main cause of the general problem with the MACI approach, ie, frequent formation of fibrocartilage (containing collagen type I and poorly sustainable clinically) instead of hyaline cartilage.^48^ This problem is thought to be caused by the partial dedifferentiation of chondrocytes during their expansion.^7,49,50^ Thus, inhibition of Notch signaling during expansion may reduce the production of fibrocartilage in connection with MACI.

Importantly, we observed that inhibition of Notch signaling is beneficial not only for the expansion of artSPCs, but also for articular chondrocytes. Since articular chondrocytes are physiologically non-proliferative, they probably have to partially dedifferentiate before being able to expand on plastic^51^ and blocking of Notch signaling might potentially direct this de-differentiation toward chondroprogenitors. The role of Notch signaling in the de-differentiation of artSPCs and chondrocytes is exemplified further by the observation that activation of Notch by Jagged 1 during the expansion phase results in the total loss of the ability of these cells to form cartilage.

De-differentiation of chondrocytes or, more generally, their plasticity is rather remarkable. Approximately 40%-60% of the hypertrophic chondrocytes in the growth plate transdifferentiate into osteoblasts,^52^ with a recent report suggesting that this process involves de-differentiation followed by subsequent differentiation toward osteogenic, adipogenic, and other stromal lineages.^33^ Indeed, the transition from hypertrophic chondrocytes to other lineages includes a cell stage characterized by the expression of numerous markers specific to skeletal stem cells.^33^

Our unexpected observation that articular chondrocytes, even those obtained from elderly patients, are capable of differentiating into osteogenic and adipogenic lineages in culture is consistent with the extensive plasticity of chondrocytes and also indicates that cultured chondrocytes undergo dedifferentiation. Interestingly, such an ability of aged human deep zone articular chondrocytes to differentiate toward osteo- and adipolineages in vitro has been reported but was interpreted as potential contamination by multipotent progenitors.^25^ Our current observations including those with lineage tracing suggest rather an intrinsic plasticity of articular chondrocytes. Of note, at least fetal/neonatal articular chondrocytes give rise to bone cells in vivo,^13^ again exemplifying their remarkable plasticity. Thus, although the mechanism(s) underlying de-differentiation of chondrocytes in vivo are currently poorly understood, the plasticity of these cells *ex vivo* appears to be tightly controlled by Notch signaling, which may have important implications for cartilage regeneration.

The role of the Notch pathway in determining the fate of embryonic cells is well known.^53^ For example, Notch participates actively in skeletal development by controlling progression from less toward more differentiated cells of both the osteogenic and chondrogenic lineages in vivo.^54–57^ In cultures of limb bud pellets inhibition of the Notch pathway promotes chondrogenic differentiation.^58^ Activation of this same pathway in chondrogenic ATDC5 cells inhibits their differentiation toward chondrocytes.^59,60^ Thus, the ability of Notch signaling to modulate chondrogenic potential during the expansion of artSPCs observed here is consistent with the general Notch-dependent regulation of cell fate. At the same time, the role of Notch in development may not reflect its role in the pathophysiology of adult articular cartilage.

Members of the Notch signaling pathway are expressed differentially in the different zones of articular cartilage, with Notch1 being expressed exclusively in the superficial layer, where the artSPCs are located, and Notch2 being expressed more uniformly throughout the articular cartilage.^11,31,61^ The Notch agonists Jagged1 and Delta4 have been detected in human articular cartilage, predominantly in the superficial zone.^31^ Furthermore, the level of expression of both Notch receptors and ligands increases dramatically in connection with the development of osteoarthritis,^31,61,62^ suggesting a role for this pathway in pathological processes involving cartilage. Any such role is likely to be negative since genetic activation of the Notch pathway in cartilage for a prolonged period causes degeneration of this tissue in a manner dependent on IL6.^63^ At the same time, ablation of the key downstream component of the Notch pathway, RBP-Jk, protects the joint from surgery-induced osteoarthritis^62^ but results in age-dependent osteoarthritis.^64^ It appears plausible that surgical induction of osteoarthritis activates Notch signaling throughout the cartilage,^62^ but in mice lacking RBP-Jk, this elevation of Notch activity is abrogated. Probably a certain low level of Notch activity is required for maintenance of the physiological homeostasis of cartilage and ablation of Notch may disrupt this homeostasis and cause the degeneration of cartilage with age.^64^

In vitro observations concerning the role of the Notch pathway in the regulation of chondrocytes and chondroprogenitors are rather confusing. Although blocking Notch signaling with DAPT has been reported to impair the proliferation of chondrocytes,^61^ as well as the formation of colonies by artSPCs that were isolated via adherence to fibronectin,^11^ such blockage has also been reported to improve the proliferation of chondrocytes.^65^ The latter observation agrees with our present findings.

One can only speculate on the potential reasons for such discrepancies, which may involve differences in the dose and duration of treatment, preparation of cells (exemplified here by the difference in the extent of expansion of artSPCs isolated by MACS or FACS upon DAPT treatment), epigenetic status of the cells obtained, and many other factors as well. However, we would like to emphasize that the experiments documented here were reproduced in two different laboratories by 3 independent researchers (L.L., A.D.K., and A.P.U). Thus, minor variations in conditions and handling can probably be excluded.

In summary, we conclude that the Notch pathway is a primary regulator of chondrogenic potential during culturing of articular chondrocytes or artSPCs. Inhibition of this pathway allows rapid expansion of these cells with simultaneous preservation of their ability to form hyaline cartilage.

## Experimental Procedures

### Animals

All animal experiments were pre-approved by the bioethics committee of Sechenov University (Moscow, Russian Federation) and the Ethical Committee on Animal Experiments (Stockholm North Committee/Norra Djurförsöksetiska Nämd, Stockholm, Sweden). Mice were used on postnatal day (P) 3-7. For experiments not involving transgenic strains, as well as for the collection of cartilage, C57Bl6 mice were used. Col2-CreER mice were obtained from Susanne Mackem^66^; the Rosa26-Confetti animals from Snippert et al.^67^; and the Prg4CreER strain from Andrew Lassar.^12^ Prior to tissue collection mice were euthanized by an overdose of isoflurane.

### Human Samples

Following the provision of written informed consent, human articular cartilage was obtained from patients in connection with knee surgery. The surgeries were conducted due to osteoarthritis of the joint leading to total knee replacement. Cartilage from 5 female patients with one-sided (either lateral or medial) osteoarthritis was used for experiments. Patients were 46, 66, 66, 69, and 71 years of age at the time of cartilage collection. Only intact cartilage was collected for the experiments.

### Cell Isolation

Cartilage from the knees of mice 5 ± 2 days old was dissected out into ice-cold medium (DMEM/F-12-HEPES containing 0.1 mg/mL gentamycin) and as much of the ligaments, tendons, and other tissue surrounding the cartilage as possible gently removed under a stereomicroscope. The cartilage was subsequently incubated with 0.15% collagenase type II for 2-5 h at 37 °C on a roller. For every experiment, cells were isolated from one litter (5-6 mice).

Human samples were washed in sterile PBS containing 0.1 mg/mL gentamycin and, thereafter, intact cartilage was dissected out, washed in dissection medium, cut into 1-2 mm fragments, and incubated in 0.1% collagenase type II overnight at 37 °C on a roller.

### Magnetic-Activated Cell Sorting (MACS)

Isolated murine cells were passed through a filter (40 um) into MACS buffer (phosphate-buffered saline (PBS), 2% FBS, 1 mM EDTA) and then subjected to MACS as described by Gagliardi and colleagues (2018). In brief, the cells were incubated with magnetic beads covered with an antibody against CD73 conjugated with PE (diluted 1:10, clone TY/11.8, Biolegend) at 4 °C for 15 minutes. After subsequent washing with MACS buffer, MicroBeads (Miltenyi Biotec) were mixed with the cell suspension for 15 minutes at 4 °C. Following 3 washes, CD73^high^ cells were then collected on a magnetic platform.

### Flow Cytometry and Fluorescence-Activated Cell Sorting (FACS)

To test their purity, CD73^high^ cells obtained by MACS were fixed and permeabilized in accordance with the manufacturer’s instructions (BD Bioscience). Intracellular staining for Sox9 (HPA001758 Sigma) was also performed as described by BD Bioscience and the data acquired employing LSRII software (BD Bioscience).

For the performance of FACS, cells were stained with the same CD73 antibody (diluted 1:100 and conjugated with PE or APC). Five hundred thousand human cells were stained with CD29 antibodies conjugated with PE (Miltenyi Biotec, clone TS2/16, 1:100, 100 uL per aliquot). Murine cells were sorted on a FACSAria III column (BD Bioscience) and human cells on a Sony SH800 device. Flow cytometric data were analyzed with the FlowJo software (version 10, TreeStar).

### Analysis of Colony Formation and Size

CD73^high^ cells obtained from Prg4-CreERT2 mice^12^ crossed with R26R-Confetti mice^67^ as described above were seeded at low density (250 cells/cm^2^) and cultured for 12 days. Thereafter, colony number and size were evaluated by staining with H&E as well as directly by fluorescence microscopy. Mice were pulsed with tamoxifen on day 3 ± 1 and cells were collected 2-3 days later.

### Determination of the Cells’ Differentiation Potential

Adipogenic and osteogenic differentiation were assessed as described recently.^68^ In brief, when isolated cells at first passage had reached 80% confluence, either adipogenic or osteogenic differentiation medium was added for 21 days, following which the cells were stained with Oil Red O or alizarin red, respectively.

### Assay of Chondrogenic Differentiation (Pellet Cultures)

After 3 passages, 150 000 cells from each group were centrifuged for 10 minutes at 400 *g* and 4 °C in 1.5 mL Eppendorf tubes and then allowed to aggregate for 2 h at 37 °C in expansion medium (10% FBS, 1% PenStrep, 5 ug/mL ascorbic acid in DMEM). Thereafter, the medium was carefully changed to chondrogenic medium (100 uM 2-ME, 2mM pyruvate, 0.35 mM L-proline, 1% ITS+3, 1% PenStrep, 5 ug/mL ascorbic acid, 0.1 uM dexamethasone, 10 ng/mL TGFb3 in DMEM^68^). Two days later, the pellets obtained were transferred to a multiple-well dish and cultured for 21 days.

### Cell Culture

Following MACS, CD73^high^ cells were seeded onto plates (5000 cells/cm^2^) and expanded in presence of either LiCl, which stimulates Wnt/b-catenin (10 mM, Sigma-Aldrich), the Wnt/b-catenin inhibitor XAV-939 (10 uM, Selleckchem), the Jagged1, which is a Notch ligand (10 ug/mL, R&D Systems), the Notch inhibitor DAPT (10 uM, Sigma-Aldrich), or FGF2 (10 ng/mL) for 3 passages. For analysis of the role of adhesion molecules, Petri-dishes were covered with fibronectin (Fi) (3 ug/cm^2^), while the control dishes received only PBS. The medium was replaced with fresh medium supplemented with activators/inhibitors once every 3 days and in connection with each passage, 10 000 cells were collected for analysis of gene expression.

The procedures for delivery of active Jagged1 to cells in culture have been described previously.^69^ In brief, 20 ug/mL Fc-specific human IgG (Sigma-Aldrich) diluted in PBS was incubated in the culture dishes at room temperature for 2 h. Thereafter, 10 ug/mL of either recombinant rat Jagged1 fused to human Fc (R&D Systems) or human Fc control protein (R&D Systems) dissolved in PBS was added to the dishes pre-coated with Fc-specific human IgG and incubated at 4 °C overnight. This medium was removed before seeding the cells.

After FACS, the cells were seeded (28 000 cells/cm^2^) and subsequently treated with DAPT, Jagged1, and fibronectin as described above. The higher density, as compared with MASC, was used to facilitate cell survival upon the FACS isolation.

### Analysis of Gene Expression

Total RNA was extracted from cells in the 3rd passage with the RNeasy Micro Kit (QIAGEN) and reverse-transcribed into cDNA utilizing the PrimeScript RT Reagent Kit (TAKARA). The SYBR GREEN QuantiTect RT-PCR Kit (Thermo Fisher) was used for real-time PCR analysis, employing the primers listed in Table 1. The levels of mRNA encoding Prg4, GDF5, DKK3, CD73, Col1a1, Col2a1, and SOX9 were normalized to the housekeeping gene Hprt employing the 2-ΔCt formula. The levels of Notch1, Noch2, Notch3, Notch4, Hes1, and Hey1 expression were normalized to the corresponding levels on P0, and the calculation formula 2-ΔΔCt used.

**Table 1.** 5ʹ-3ʹ sequences of the primers used.

Genes	Gene full name	Forward	Reverse
*Col2a1*	Collagen type II alpha 1 chain	GCCAGGATGCCCGAAAATTAG	GTCACCTCTGGGTCCTTGTTC
*Sox9*	SRY-box transcription factor 9	ACGGCTCCAGCAAGAACAAG	TTGTGCAGATGCGGGTACTG
*Col1a1*	Collagen type I alpha 1 chain	TTCTGGTCCTCGTGGTCTCC	GACCCATTGGACCTGAAGCG
*Prg4*	Proteoglycan 4	CAAAGGACGCTGCTTCGAGT	GGTGCAGTCTTTGGAGGTGG
*Dkk3*	Dickkopf WNT signaling pathway inhibitor 3	TCAGGAGGAAGCTACGCTCAATG	TCTCCGTGCTGGTCTCATTGTG
*Gdf5*	Growth differentiation factor 5	CGGACTGTAACCCCAAAAGGA	TCCGTAAGATCCGCAGTTCAG
*CD73*	5ʹ-Nucleotidase Ecto	CAAATCCCACACAACCACTG	TGCTCACTTGGTCACAGGAC
*Notch1*	Notch receptor 1	CCCACTGTGAACTGCCCTAT	CCCCATTCTTGCAGTTGTT
*Notch2*	Notch receptor 2	GAACCGTGGAGATGAACGAGAC	CAGAGGCTGGGAAAGGATGATAGG
*Notch3*	Notch receptor 3	TGAGTGTCCAGCTGGCTATG	CACAGGTGCCATTGTGTAGG
*Notch4*	Notch receptor 4	AAGCGACACGTACGAGTCTGG	ATAGTTGCCAGCTACTTGTGG
*Hes1*	Hes family BHLH transcription factor 1	CCAGCCAGTGTCAACACGA	AATGCCGGGAGCTATCTTTCT
*Hey1*	Hes related family BHLH transcription factor with YRPW motif 1	TCTCCCTTCACCTCACTGCT	CACGCCACTATGCTCAATGT
*Hprt*	Hypoxanthine phosphoribosyltransferase 1	ACAGGCCAGACTTTGTTGGA	ACTTGCGCTCATCTTAGGCT

### Immunofluorescent Staining and Staining With SafraninO and Fast Green

Chondropellets were fixed with 10% neutral buffered formalin for 6 h, embedded in OCT, and cut into 10-um sections. Primary antibodies against Sox9 (1:200, HPA001758 Sigma), collagen I (1:200, SAB4500362 Sigma for human material and CSI 008-01-02 Invitrogen for mouse), collagen II (1:200, PA1-26206 Invitrogen), Runx2 (1:500, ab23981 Abcam), Mef2c (1:300 HPA005533 Sigma), and Osx (1:200 PA5-40411 Invitrogen) were used. Antigen retrieval in the case of Mef2c, Osx, and Runx2 was achieved with boiling DAKO buffer; for Sox9 with 0.1% trypsin for 30 minutes at room temperature; and for Col I and Col II with 0.5% pepsin in 5 mM HCl for 20 minutes at 37 °C. Secondary antibodies conjugated with various fluorophores (1:400, Jackson lab) were applied for 1.5 h at room temperature.

Samples of human articular cartilage were fixed with 10% neutral buffered formalin for 6 h. In the case of staining for CD29 (1:100) antigen retrieval was achieved with boiling DAKO buffer followed by 0.5% pepsin in 5 mM HCl for 20 minutes at 37 °C.

For immunocytochemical staining, antibodies against Osx (1:400 PA5-40411 Invitrogen) or Perilipin-1 (1:400 9349 Cell Signaling Technology) were used. The cells were fixed with 10% neutral buffered formalin for 30 min. Primary antibodies were applied for 3 h and secondary antibodies for 30 min, in both cases at room temperature.

Imaging was performed with a confocal Zeiss LSM880 fluorescence microscope. Positive cells were counted, and the surface was reconstructed with the Imaris 7.4.2 or ImageJ software.

The protocol for staining with safranin O & fast green has been described elsewhere.^70,71^

### Statistical Analysis

All values presented are means ± SD. The normal distribution of all data was confirmed utilizing the Shapiro-Wilk test. The unpaired Student’s t test was used to compare 2 groups and one-way ANOVA with Tukey’s multiple-comparison test for the comparison of multiple groups. For clarity, the figures illustrate data relative to the appropriate controls; however, for statistical analysis, the control and experimental values were compared.

The standard deviation for relative change (*Δ*%) was calculated using the formula


$$\begin{array}{l} SD=\;\;\;\sqrt{\frac{\overset{n}{\underset{i=1}{\sum }}X_{i}^{2}}{n}\times \frac{\overset{n}{\underset{i=1}{\sum }}{\left(\frac{1}{Y_{i}}\right)}^{2}}{n}-{\left(\frac{\overset{n}{\underset{i=1}{\sum }}X_{i}}{n}\times \frac{\overset{n}{\underset{i=1}{\sum }}\frac{1}{Y_{1}}}{n}\right)}^{2}}\; \end{array}$$

where *X* is the experimental values, *Y* is the control values, and *n* is the number of data points.

The standard deviation for absolute difference (%-%, Fig. 3, P-R) were calculated using the formula


$$\begin{array}{l} SD=\sqrt{{SD}_{X}^{2}+{SD}_{Y}^{2}} \end{array}$$

where SD_*x*_ and SD_*y*_ are the standard deviations for the experimental and control groups, respectively.

## Supplementary Material

sxad031_suppl_Supplementary_Figures

## Data Availability

The data that support the findings of this study are available from the corresponding author upon reasonable request.

## References

[1] Mallein-Gerin F , Kosher RA, Upholt WB, Tanzer ML. Temporal and spatial analysis of cartilage proteoglycan core protein gene expression during limb development by in situ hybridization. Dev Biol. 1988;126(2):337-345. 10.1016/0012-1606(88)90144-3. 3280365

[2] Jay GD , Tantravahi U, Britt DE, Barrach HJ, Cha CJ. Homology of lubricin and superficial zone protein (SZP): products of megakaryocyte stimulating factor (MSF) gene expression by human synovial fibroblasts and articular chondrocytes localized to chromosome 1q25. J Orthop Res. 2001;19(4):677-687. 10.1016/S0736-0266(00)00040-1. 11518279

[3] Brittberg M , Lindahl A, Nilsson A, Ohlsson C, Isaksson O, Peterson L. Treatment of deep cartilage defects in the knee with autologous chondrocyte transplantation. N Engl J Med. 1994;331(14):889-895. 10.1056/NEJM199410063311401 8078550

[4] Drobnic M , Kregar-Velikonja N, Radosavljevic D, et al. The outcome of autologous chondrocyte transplantation treatment of cartilage lesions in the knee. Cell Mol Biol Lett. 2002;7(2):361-363. 12097986

[5] Kon E , Filardo G, Di Martino A, Marcacci M. ACI and MACI. J Knee Surg. 2012;25(1):17-22. 10.1055/s-0031-1299651. 22624243

[6] von der Mark K , Gauss V, von der Mark H, Müller P. Relationship between cell shape and type of collagen synthesised as chondrocytes lose their cartilage phenotype in culture. Nature. 1977;267(5611):531-532. 10.1038/267531a0. 559947

[7] Minegishi Y , Hosokawa K, Tsumaki N. Time-lapse observation of the dedifferentiation process in mouse chondrocytes using chondrocyte-specific reporters. Osteoarthr Cartil. 2013;21(12):1968-1975. 10.1016/j.joca.2013.09.004 24091160

[8] Chen Y , Yu Y, Wen Y, et al. A high-resolution route map reveals distinct stages of chondrocyte dedifferentiation for cartilage regeneration. Bone Res. 2022;10(38):1-16. 35477573 10.1038/s41413-022-00209-w PMC9046296

[9] Anderson DE , Markway BD, Weekes KJ, McCarthy HE, Johnstone B. Physioxia promotes the articular chondrocyte-like phenotype in human chondroprogenitor-derived self-organized tissue. Tissue Eng Part A. 2018;24(3-4):264-274. 10.1089/ten.TEA.2016.0510. 28474537 PMC5792248

[10] Barlič A , Drobnič M, Maličev E, Kregar-Velikonja N. Quantitative analysis of gene expression in human articular chondrocytes assigned for autologous implantation. J Orthop Res. 2008;26(6):847-853. 10.1002/jor.20559. 18186131

[11] Dowthwaite GP , Bishop JC, Redman SN, et al. The surface of articular cartilage contains a progenitor cell population. J Cell Sci. 2004;117(Pt 6):889-897. 10.1242/jcs.00912. 14762107

[12] Kozhemyakina E , Zhang M, Ionescu A, et al. Identification of a Prg4-expressing articular cartilage progenitor cell population in mice. Arthritis Rheumatol (Hoboken, NJ). 2015;67(5):1261-1273. 10.1002/art.39030 PMC4414823 25603997

[13] Li L , Newton PT, Bouderlique T, et al. Superficial cells are ­self-renewing chondrocyte progenitors, which form the articular cartilage in juvenile mice. FASEB J. 2017;31(3):1067-1084. 10.1096/fj.201600918R. 27965322 PMC5295727

[14] Decker RS. Articular cartilage and joint development from embryogenesis to adulthood. Semin Cell Dev Biol. 2017;62:50-56. 10.1016/j.semcdb.2016.10.005. 27771363 PMC6041433

[15] Yasuhara R , Ohta Y, Yuasa T, et al. Roles of β-catenin signaling in phenotypic expression and proliferation of articular cartilage superficial zone cells. Lab Investig. 2011;91(12):1739-1752. 10.1038/labinvest.2011.144. 21968810 PMC3759358

[16] Feng C , Chan WCW, Lam Y, et al. Lgr5 and Col22a1 mark progenitor cells in the lineage toward juvenile articular chondrocytes. Stem Cell Rep. 2019;13(4):713-729. 10.1016/j.stemcr.2019.08.006. PMC6829785 31522976

[17] Kurenkova AD , Medvedeva EV, Newton PT, Chagin AS. Niches for skeletal stem cells of mesenchymal origin. Front Cell Dev Biol. 2020;8:592. 10.3389/fcell.2020.00592. 32754592 PMC7366157

[18] Spradling A , Drummond-Barbosa D, Kai T. Stem cells find their niche. Nature. 2001;414(6859):98-104. 10.1038/35102160. 11689954

[19] Shahbazi MN , Siggia ED, Zernicka-Goetz M. Self-organization of stem cells into embryos: a window on early mammalian development. Science. 2019;364(6444):948-951. 10.1126/science.aax0164. 31171690 PMC8300856

[20] Chijimatsu R , Saito T. Mechanisms of synovial joint and articular cartilage development. Cell Mol Life Sci. 2019;76(20):3939-3952. 10.1007/s00018-019-03191-5. 31201464 PMC11105481

[21] Hartmann C , Tabin CJ. Wnt-14 plays a pivotal role in inducing synovial joint formation in the developing appendicular skeleton. Cell. 2001;104(3):341-351. 10.1016/s0092-8674(01)00222-7. 11239392

[22] Zhu M , Tang D, Wu Q, et al. Activation of β-catenin signaling in articular chondrocytes leads to osteoarthritis-like phenotype in adult β-catenin conditional activation mice. J Bone Miner Res. 2009;24(1):12-21. 10.1359/jbmr.080901. 18767925 PMC2640321

[23] Nalesso G , Sherwood J, Bertrand J, et al. WNT-3A modulates articular chondrocyte phenotype by activating both canonical and noncanonical pathways. J Cell Biol. 2011;193(3):551-564. 10.1083/jcb.201011051. 21536751 PMC3087013

[24] Xie Y , Zinkle A, Chen L, Mohammadi M. Fibroblast growth factor signalling in osteoarthritis and cartilage repair. Nat Rev Rheumatol. 2020;16(10):547-564. 10.1038/s41584-020-0469-2. 32807927

[25] Williams R , Khan IM, Richardson K, et al. Identification and clonal characterisation of a progenitor cell sub-population in normal human articular cartilage. PLoS One. 2010;5:e13246. 10.1371/journal.pone.0013246. 20976230 PMC2954799

[26] Felsenthal N , Zelzer E. Mechanical regulation of musculoskeletal system development. Development. 2017;144(23):4271-4283. 10.1242/dev.151266. 29183940 PMC6514418

[27] Ogawa H , Kozhemyakina E, Hung H-H, Grodzinsky AJ, Lassar AB. Mechanical motion promotes expression of Prg4 in articular cartilage via multiple CREB-dependent, fluid flow shear stress-induced signaling pathways. Genes Dev. 2014;28(2):127-139. 10.1101/gad.231969.113. 24449269 PMC3909787

[28] Basoli V , Della Bella E, Kubosch EJ, Alini M, Stoddart MJ. Effect of expansion media and fibronectin coating on growth and chondrogenic differentiation of human bone marrow-derived mesenchymal stromal cells. Sci Rep. 2021;11(1):13089. 10.1038/s41598-021-92270-4. 34158528 PMC8219706

[29] Kalkreuth RH , Krüger JP, Lau S, et al. Fibronectin stimulates migration and proliferation, but not chondrogenic differentiation of human subchondral progenitor cells. Regen Med. 2014;9(6):759-773. 10.2217/rme.14.40. 25431912

[30] Kachroo U , Ramasamy B, Vinod E. Evaluation of CD49e as a distinguishing marker for human articular cartilage derived chondroprogenitors. Knee. 2020;27(3):833-837. 10.1016/j.knee.2020.04.002. 32317141

[31] Ustunel I , Ozenci AM, Sahin Z, et al. The immunohistochemical localization of notch receptors and ligands in human articular cartilage, chondroprogenitor culture and ultrastructural characteristics of these progenitor cells. Acta Histochem. 2008;110(5):397-407. 10.1016/j.acthis.2007.12.005. 18272209

[32] Vinod E , Johnson NN, Kumar S, et al. Migratory chondroprogenitors retain superior intrinsic chondrogenic potential for regenerative cartilage repair as compared to human fibronectin derived chondroprogenitors. Sci Rep. 2021;11:23685. 34880351 10.1038/s41598-021-03082-5 PMC8654938

[33] Long JT , Leinroth A, Liao Y, et al. Hypertrophic chondrocytes serve as a reservoir for marrow-associated skeletal stem and progenitor cells, osteoblasts, and adipocytes during skeletal development. Elife. 2022;11:e76932. 35179487 10.7554/eLife.76932 PMC8893718

[34] Xue K , Zhang X, Qi L, Zhou J, Liu K. Isolation, identification, and comparison of cartilage stem progenitor/cells from auricular cartilage and perichondrium. Am J Transl Res. 2016;8(2):732-741. 27158365 PMC4846922

[35] Carluccio S , Martinelli D, Palamà MEF, et al. Progenitor cells activated by platelet lysate in human articular cartilage as a tool for future cartilage engineering and reparative strategies. Cells. 2020;9(4):1052. 10.3390/cells9041052. 32340136 PMC7226425

[36] De Luca P , Kouroupis D, Viganò M, et al. Human diseased articular cartilage contains a mesenchymal stem cell-like population of chondroprogenitors with strong immunomodulatory responses. J Clin Med. 2019;8(4).):423-438. 30925656 10.3390/jcm8040423 PMC6517884

[37] Mabuchi Y , Okawara C, Méndez-Ferrer S, Akazawa C. Cellular heterogeneity of mesenchymal stem/stromal cells in the bone marrow. Front Cell Dev Biol. 2021;9:689366. 34295894 10.3389/fcell.2021.689366 PMC8291416

[38] Wang Z , Li X, Yang J, et al. Single-cell RNA sequencing deconvolutes the in vivo heterogeneity of human bone marrow-derived mesenchymal stem cells. Int J Biol Sci. 2021;17(15):4192-4206. 10.7150/ijbs.61950. 34803492 PMC8579438

[39] Parekh C , Crooks GM. Critical differences in hematopoiesis and lymphoid development between humans and mice. J Clin Immunol. 2013;33(4):711-715. 10.1007/s10875-012-9844-3. 23274800 PMC3633618

[40] Zhang Z , He J-W, Fu W-Z, Zhang C-Q, Zhang Z-L. Calcification of joints and arteries: second report with novel NT5E mutations and expansion of the phenotype. J Hum Genet. 2015;60(10):561-564. 10.1038/jhg.2015.85. 26178434

[41] Shkhyan R , Lee S, Gullo F, et al. Genetic ablation of adenosine receptor A3 results in articular cartilage degeneration. J Mol Med (Berl). 2018;96(10):1049-1060. 10.1007/s00109-018-1680-3. 30088034 PMC8493638

[42] McCoy AM. Animal models of osteoarthritis: comparisons and key considerations. Vet Pathol. 2015;52(5):803-818. 10.1177/0300985815588611. 26063173

[43] Goldring MB , Otero M. Inflammation in osteoarthritis. Curr Opin Rheumatol. 2011;23(5):471-478. 10.1097/BOR.0b013e328349c2b1. 21788902 PMC3937875

[44] Seok J , Warren HS, Cuenca AG, et al; Inflammation and Host Response to Injury, Large Scale Collaborative Research Program. Genomic responses in mouse models poorly mimic human inflammatory diseases. Proc Natl Acad Sci USA. 2013;110(9):3507-3512. 10.1073/pnas.1222878110. 23401516 PMC3587220

[45] Malfait A-M , Little CB. On the predictive utility of animal models of osteoarthritis. Arthritis Res Ther. 2015;17(1):225. 10.1186/s13075-015-0747-6. 26364707 PMC4568581

[46] Oo WM , Hunter DJ. Disease modification in osteoarthritis: are we there yet? Clin Exp Rheumatol. 2019;37(Suppl 120):135-140. 31621568

[47] Behrens P , Bitter T, Kurz B, Russlies M. Matrix-associated autologous chondrocyte transplantation/implantation (MACT/MACI)-5-year follow-up. Knee. 2006;13(3):194-202. 10.1016/j.knee.2006.02.012. 16632362

[48] Gille J , Behrens P, Schulz AP, Oheim R, Kienast B. Matrix-associated autologous chondrocyte implantation: a clinical ­follow-up at 15 years. Cartilage. 2016;7(4):309-315. 10.1177/1947603516638901. 27688839 PMC5029570

[49] Duan L , Ma B, Liang Y, et al. Cytokine networking of chondrocyte dedifferentiation in vitro and its implications for cell-based cartilage therapy. Am J Transl Res. 2015;7(2):194-208. 25901191 PMC4399086

[50] Schulze-Tanzil G. Activation and dedifferentiation of chondrocytes: implications in cartilage injury and repair. Ann Anat. 2009;191(4):325-338. 10.1016/j.aanat.2009.05.003. 19541465

[51] Melero-Martin JM , Al-Rubeai M. In vitro expansion of chondrocytes. In: Ashammakhi N., ed. RR& EC©, Top. Tissue Engineering, Oulu University, 2007:1-37.

[52] Zhou X , von der Mark K, Henry S, et al. Chondrocytes transdifferentiate into osteoblasts in endochondral bone during development, postnatal growth and fracture healing in mice. PLoS Genet. 2014;10(12):e1004820. 10.1371/journal.pgen.1004820. 25474590 PMC4256265

[53] Artavanis-Tsakonas S , Rand MD, Lake RJ. Notch signaling: cell fate control and signal integration in development. Science. 1999;284(5415):770-776. 10.1126/science.284.5415.770. 10221902

[54] Zanotti S , Canalis E. Notch and the skeleton. Mol Cell Biol. 2010;30(4):886-896. 10.1128/MCB.01285-09. 19995916 PMC2815558

[55] Hilton MJ , Tu X, Wu X, et al. Notch signaling maintains bone marrow mesenchymal progenitors by suppressing osteoblast differentiation. Nat Med. 2008;14(3):306-314. 10.1038/nm1716. 18297083 PMC2740725

[56] Dong Y , Jesse AM, Kohn A, et al. RBPjκ-dependent notch signaling regulates mesenchymal progenitor cell proliferation and differentiation during skeletal development. Development. 2010;137(9):1461-1471. 10.1242/dev.042911. 20335360 PMC2853848

[57] Mead TJ , Yutzey KE. Notch pathway regulation of chondrocyte differentiation and proliferation during appendicular and axial skeleton development. Proc Natl Acad Sci USA. 2009;106(34):14420-14425. 10.1073/pnas.0902306106. 19590010 PMC2732875

[58] Fujimaki R , Toyama Y, Hozumi N, Tezuka K-ichi. Involvement of Notch signaling in initiation of prechondrogenic condensation and nodule formation in limb bud micromass cultures. J Bone Miner Metab. 2006;24(3):191-198. 10.1007/s00774-005-0671-y. 16622731

[59] Watanabe N , Tezuka Y, Matsuno K, et al. Suppression of differentiation and proliferation of early chondrogenic cells by Notch. J Bone Miner Metab. 2003;21(6):344-352. 10.1007/s00774-003-0428-4. 14586790

[60] Xu W , Wang Y, Zhao H, et al. Delta-like 2 negatively regulates chondrogenic differentiation. J Cell Physiol. 2018;233(9):6574-6582. 10.1002/jcp.26244. 29057471 PMC13482451

[61] Karlsson C , Jonsson M, Asp J, et al. Notch and HES5 are regulated during human cartilage differentiation. Cell Tissue Res. 2007;327(3):539-551. 10.1007/s00441-006-0307-0. 17093926

[62] Hosaka Y , Saito T, Sugita S, et al. Notch signaling in chondrocytes modulates endochondral ossification and osteoarthritis development. Proc Natl Acad Sci USA. 2013;110(5):689366. 10.1073/pnas.1207458110 PMC3562777 23319657

[63] Liu Z , Chen J, Mirando AJ, et al. A dual role for NOTCH signaling in joint cartilage maintenance and osteoarthritis. Sci Signal. 2015;8(10):2623-2633. 10.1126/scisignal.aaa3792 PMC4607068 26198357

[64] Mirando AJ , Liu Z, Moore T, et al. RBPjκ-dependent Notch signaling is required for articular cartilage and joint maintenance. Arthritis Rheum. 2013;65:n/a-n/a. 10.1002/art.38076 PMC4038327 23839930

[65] Sassi N , Gadgadi N, Laadhar L, et al. Notch signaling is involved in human articular chondrocytes de-differentiation during osteoarthritis. J Recept Signal Transduct Res. 2014;34(1):48-57. 10.3109/10799893.2013.856920. 24251351

[66] Nakamura E , Nguyen M-T, Mackem S. Kinetics of tamoxifen-regulated Cre activity in mice using a cartilage-specific CreERT to assay temporal activity windows along the proximodistal limb skeleton. Dev Dyn. 2006;235(9):2603-2612. 10.1002/dvdy.20892. 16894608

[67] Snippert HJ , van der Flier LG, Sato T, et al. Intestinal crypt homeostasis results from neutral competition between symmetrically dividing Lgr5 stem cells. Cell. 2010;143(1):134-144. 10.1016/j.cell.2010.09.016. 20887898

[68] Xiao P , Dolinska M, Sandhow L, et al. Sipa1 deficiency-induced bone marrow niche alterations lead to the initiation of myeloproliferative neoplasm. Blood Adv. 2018;2(5):534-548. 10.1182/bloodadvances.2017013599. 29514790 PMC5851419

[69] Varnum-Finney B , Wu L, Yu M, et al. Immobilization of Notch ligand, Delta-1, is required for induction of notch signaling. J Cell Sci. 2000;113(23):4313-4318. 10.1242/jcs.113.23.4313. 11069775

[70] Bouderlique T , Vuppalapati KK, Newton PT, et al. Targeted deletion of Atg5 in chondrocytes promotes age-related osteoarthritis. Ann Rheum Dis. 2016;75(3):627-631. 10.1136/annrheumdis-2015-207742. 26438374 PMC4789686

[71] Kaul R , O’Brien MH, Dutra E, et al. The effect of altered loading on mandibular condylar cartilage. PLoS One. 2016;11(7):e0160121. 10.1371/journal.pone.0160121. 27472059 PMC4966927

