# Supplementary material for: Notch Signaling Regulates the Chondrogenic Potential of Both Articular Chondrocytes and Their Progenitors During Expansion
Source: Stem Cells. 2023 Apr 21;41(6):658–71. doi: 10.1093/stmcls/sxad031 (PMC10267697; doi:10.1093/stmcls/sxad031)
Supplement: sxad031_suppl_Supplementary_Figures [file sxad031_suppl_supplementary_figures.pdf]

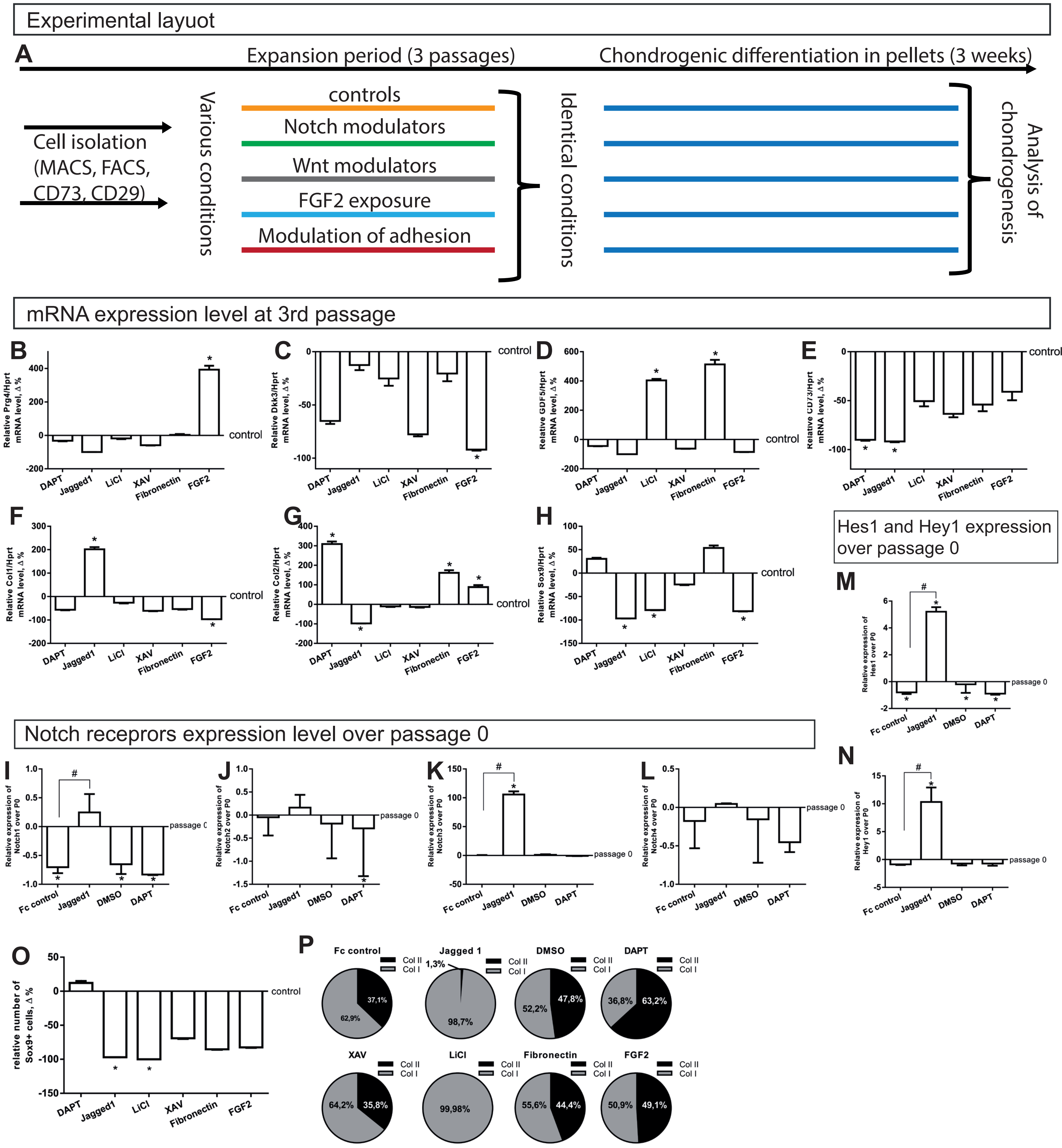

Figure S1. Characterization of the expression of various markers by murine artSPCs cultured under different conditions.

(A)The experimental layout. The relative levels of mRNA encoding (B) Prg4, (C) DKK3, (D) GDF5, (E) CD73, (F) Col1a1, (G) Col2a1 and (H) SOX9 after the third passage of MACS-isolated artSPCs were compared among each group and presented in relation to the housekeeping gene Hprt. (I-N) Expression of the Notch receptors 1-4 (I) -1, (J) -2, (K) -3, and (L) -4 and the response genes of the Notch pathway (M) Hes1 and (N) Hey1. Quantification of (O) Sox9 and (P) collagens type I and II in the pellets. All values are presented relative to those of the corresponding controls (see methods). These data are collected from 3 independent experiments. The values presented are the means±SD for 3 independent experiments. * $p<0.05$

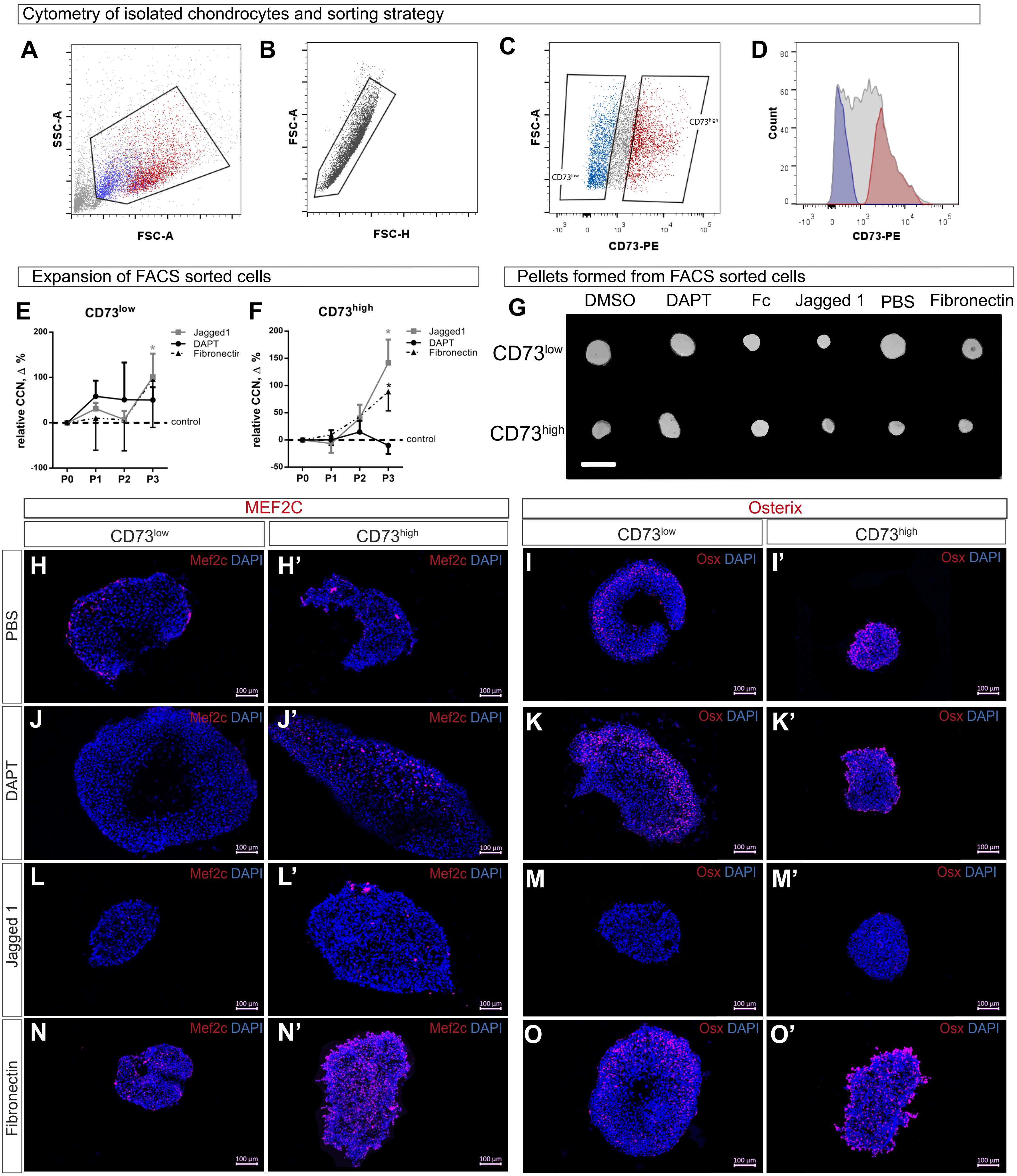

Figure S2. Additional characterization of murine artSPCs and chondrocytes.
(A-C) Gating procedure for the separation of CD73^{high} artSPCs and CD73^{low} chondrocytes by FACS and (D) overlay of the fluorescence histograms of the CD73^{low} and CD73^{high} populations of cells isolated. (E and F) The expansion rate of CD73^{low} (E) and CD73^{high} (F) cells after FACS sorting was assessed in the presence of Jagged1, DAPT, or fibronectin in relation to the corresponding controls. (G) The formation of chondrogenic pellets of different sizes from the same number of cells after 21 days of culture. Cells were expanded in the presence of different factors but underwent chondrogenic differentiation in identical conditions. Immunostaining of pellets for (H-N´) Mef2c marker of chondrocyte hypertrophy (red) and (I-O´) osterix marker (Osx) of osteogenic differentiation (red). Nuclei were stained blue with DAPI. These images are representative of 4 independent experiments. The values shown are means ± standard deviations for 3 independent experiments. *$p<0.05$
FSC-A = Forward Scatter Area; FSC-H = Forward Scatter Height; SSC-A = Side Scatter Area; PE = phycoerythrin

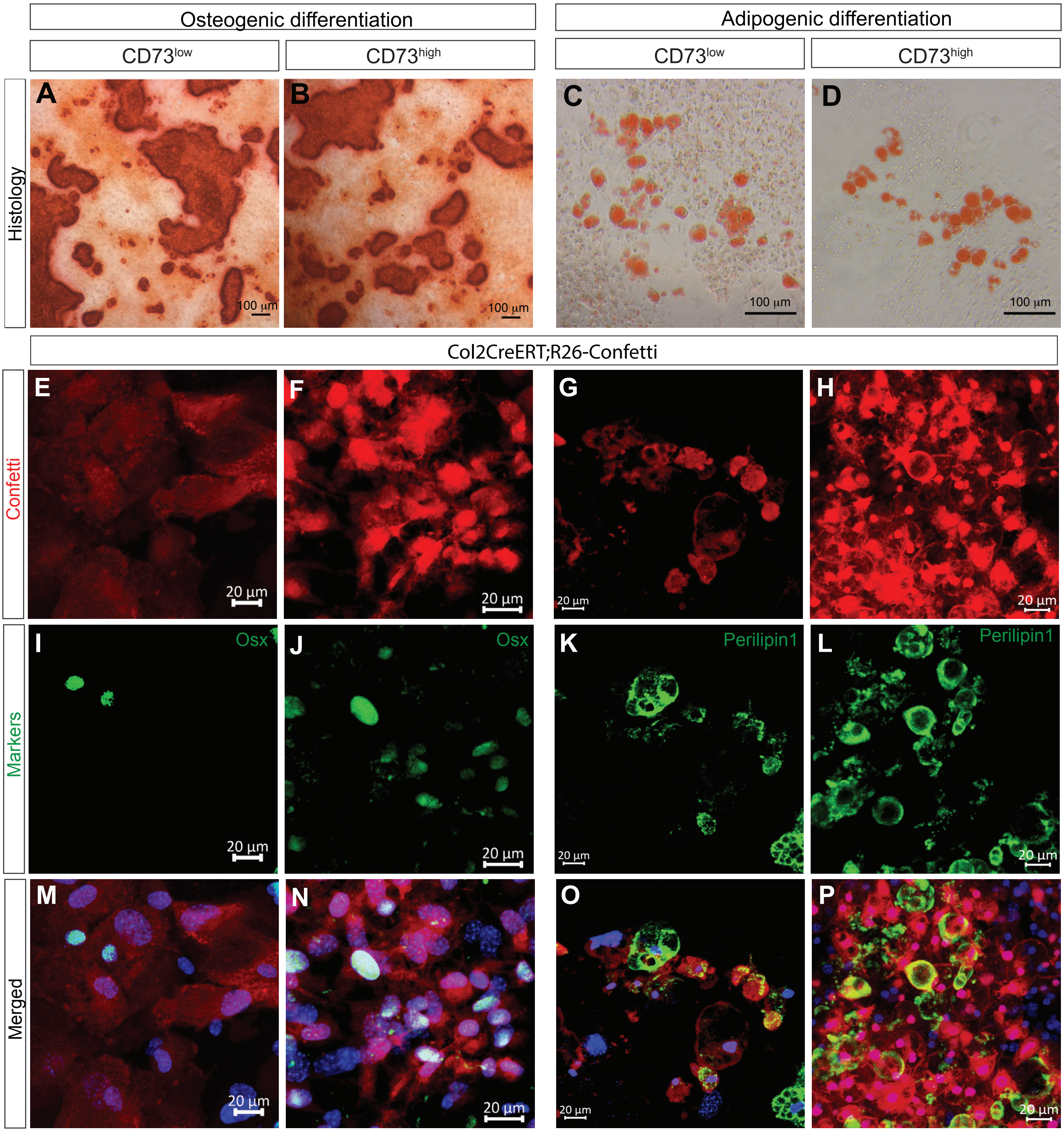

Figure S3. Differentiation of murine artSPCs and chondrocytes into the osteo- and adipogenic lineages. (A-D) CD73^{high} and CD73^{low} cells were isolated by FACS from C57Bl mice and subjected to either (A, B) osteogenic or (C, D) adipogenic differentiation. Formation of mineralized tissue was visualized by alizarin red staining (A, B) whereas the formation of adipocytes by Oil red O (C, D). (E-O) Col2-CreERT:R26R-Confetti mice were pulsed with tamoxifen at postnatal day 3 to genetically label chondrocytes *in vivo*, CD73^{high} and CD73^{low} cells were isolated by FACS 2 days later and subjected either to (E, F, I, J, M, N) osteogenic or (G, H, K, L, O, P) adipogenic differentiation. Confetti signal was pseudocolored in red (E-H), osteoblasts were immunodetected by Osterix (Osx) expression (green, I, J), and adipocytes by Perilipin1 (green, K, L). Merged confocal images exemplify cells double positive for confetti signal and either Osx (M, N) or Perilipin1 (O, P) . These images are representative of 3 independent experiments.

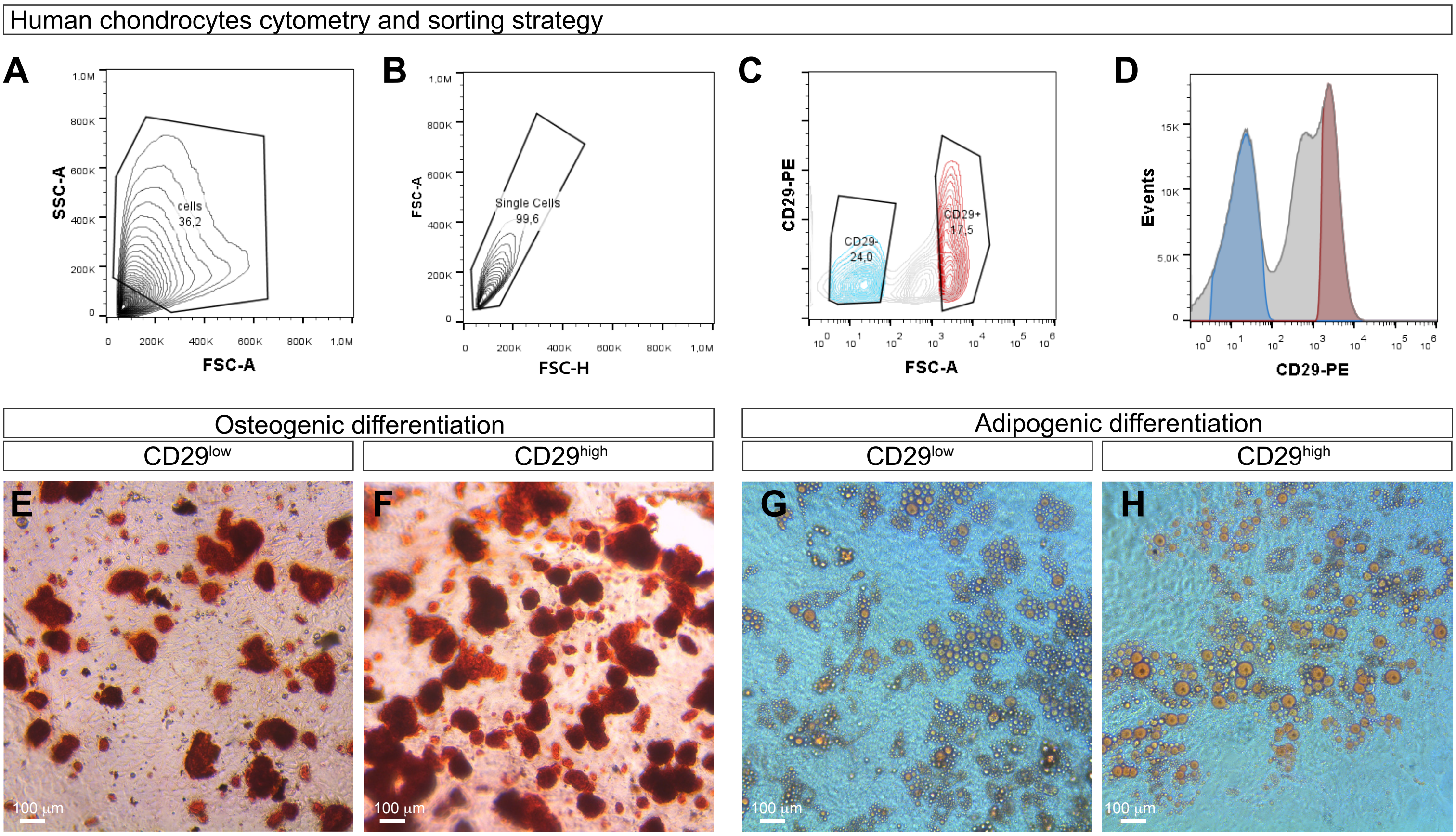

Figure S4. Isolation and differentiation of human artSPCs and chondrocytes.
(A-C) Gating strategy for the flow cytometry of human articular cartilage cells stained with CD29. (D) overlay of the fluorescence histograms of the $CD29^{low}$ and $CD29^{high}$ populations of cells isolated. (E and F) Osteogenic and (G and H) adipogenic differentiation of (E and G) $CD29^{low}$ chondrocytes and (F and H) $CD29^{high}$ artSPCs cells. These images are representative of 5 independent experiments.
